# Supplementary material for: Grape Phylloxera Genetic Structure Reveals Root–Leaf Migration within Commercial Vineyards
Source: Insects. 2021 Aug 3;12(8):697. doi: 10.3390/insects12080697 (PMC8396592; doi:10.3390/insects12080697)
Supplement: Supplementary file 1 [file insects-12-00697-s001.zip › insects-1288113-supplementary.pdf]

Table S1: List of complete MLGs; Br: Britzingen, Pf: Pfaffenweiler, Ih: Ihringen, Ba: Bahlingen. Sample taken from L: vineyard leaves, R: vineyard roots, T: thicket leaves. From hostplant Vin: V. vinifera, RH: rootstock hybrid, FR: fungus resistant variety.

MLG	n	location	hostplant	plant code	sample code	PhyIII55	PhyIII30	PhyIII36	DV8	Dvit6	DVSSR4	DV4
1	1	Br L	Vin	5/139	Br1	127:130	132:132	195:201	143:147	202:202	251:251	210:222
2	1	Br L	Vin	5/139	Br3	127:130	132:132	195:201	143:145	202:205	251:251	210:222
3	1	Br L	Vin	4/124	Br8	124:130	132:132	195:201	143:147	202:208	241:251	222:222
4	2	Br L	Vin	4/124	Br9	127:130	132:132	195:195	143:143	202:202	241:251	219:222
		Br L	Vin	4/124	Br10							
5	1	Br L	Vin	6/127	Br11	124:127	129:132	195:207	143:147	202:202	251:253	216:222
6	2	Br L	Vin	6/127	Br12	124:127	132:132	201:207	143:145	196:202	241:241	219:222
		Br L	Vin	6/127	Br14							
7	4	Br L	Vin	6/127	Br13	124:124	132:132	195:195	143:145	205:208	251:251	219:222
		Ih R	RH	21/14	Ih410							
		Ih R	RH	21/14	Ih413							
		Ih R	RH	22/38	Ih406							
8	1	Br L	Vin	6/127	Br15	124:124	129:132	195:195	143:145	205:205	251:251	219:222
9	2	Br L	Vin	3/135	Br16	127:130	129:132	195:195	145:145	202:208	251:251	222:222
		Br L	Vin	3/135	Br20							
10	2	Br L	Vin	3/135	Br17	130:130	132:132	195:195	145:145	196:208	241:245	210:222
		Br L	Vin	3/135	Br18							
11	2	Br L	Vin	3/135	Br19	130:130	129:132	195:195	145:145	196:208	241:245	210:222
		Br R	RH	3/135	Br120							
12	5	Br L	Vin	7/122	Br21	124:130	129:129	195:195	143:145	196:205	241:253	216:222
		Br L	Vin	7/122	Br23							
		Br L	Vin	7/122	Br25							
		Br L	Vin	7/122	Br22							
		Br L	Vin	7/122	Br24							
13	5	Br L	Vin	4/86	Br26	130:130	129:132	195:198	145:145	196:205	241:251	222:222
		Br L	Vin	4/87	Br33							
		Br L	Vin	4/87	Br32							
		Br L	Vin	4/87	Br34							
		Br L	Vin	4/88	Br36							
14	10	Br L	Vin	4/86	Br27	124:127	132:132	195:195	143:145	202:208	251:251	219:222
		Br L	Vin	4/86	Br28							
		Br L	Vin	4/86	Br30							
		Br L	Vin	4/87	Br35							
		Br L	Vin	4/88	Br40							
		Br L	Vin	5/85	Br51							
		Br L	Vin	5/85	Br53							
		Br L	Vin	5/85	Br54							
		Br R	RH	4/87	Br131							
		Ih R	RH	23/47	Ih430							
15	1	Br L	Vin	4/86	Br29	124:130	129:132	195:195	145:145	208:208	241:251	210:222
16	4	Br L	Vin	4/87	Br31	127:127	132:132	195:195	143:145	202:208	251:253	210:219
		Br L	Vin	4/88	Br39							
		Br R	RH	4/88	Br137							
		Br R	RH	4/88	Br140							
17	1	Br L	Vin	4/88	Br37	124:127	129:132	195:195	143:147	202:202	251:253	216:222
18	4	Br L	Vin	4/88	Br38	130:130	129:132	195:195	143:145	202:208	251:251	222:222
		Br L	Vin	6/66	Br45							
		Br L	Vin	6/67	Br47							
		Br L	Vin	6/67	Br46							
19	3	Br L	Vin	6/66	Br41	127:127	132:132	195:195	143:145	202:208	245:245	222:222
		Br L	Vin	6/66	Br43							
		Br L	Vin	6/67	Br48							
20	1	Br L	Vin	6/66	Br42	130:130	129:132	195:195	143:145	196:208	241:251	222:222
21	2	Br L	Vin	6/66	Br44	127:130	132:132	195:195	145:147	205:208	241:251	222:222
		Br L	Vin	6/67	Br49							
22	2	Br L	Vin	5/85	Br52	124:130	129:132	195:195	145:145	202:208	251:251	222:222
		Br R	RH	5/85	Br151							
23	1	Br L	Vin	5/85	Br55	124:124	129:132	195:195	145:145	202:208	251:251	222:222
24	4	Br L	Vin	5/84	Br59	124:130	129:129	195:195	145:145	202:208	241:251	222:222
		Br L	Vin	5/84	Br56							
		Br L	Vin	5/84	Br58							
		Br L	Vin	5/84	Br60							
25	1	Br L	Vin	5/53	Br62	124:130	132:132	195:195	143:145	196:202	251:251	222:222
26	3	Br L	Vin	5/53	Br63	124:124	132:132	195:207	145:145	196:202	245:251	222:222
		Br L	Vin	5/53	Br65							
		Br L	Vin	5/53	Br64							
27	1	Br L	Vin	1/142	Br66	127:127	132:132	195:195	145:145	202:202	241:251	219:222
28	4	Br L	Vin	1/142	Br67	130:130	132:132	195:195	143:147	202:205	251:253	216:222

MLG	n	location	hostplant	plant code	sample code	PhyIII55	PhyIII30	PhyIII36	DV8	Dvit6	DVSSR4	DV4
		Br L	Vin	1/142	Br69							
		Br L	Vin	1/142	Br68							
		Br L	Vin	1/142	Br70							
29	2	Br L	Vin	1/79	Br72	124:127	132:132	195:201	145:145	202:208	241:251	210:219
		Br L	Vin	1/79	Br74							
30	1	Br L	Vin	1/79	Br73	127:130	132:132	195:207	143:145	202:208	251:251	222:222
31	1	Br L	Vin	1/79	Br75	124:130	132:132	195:195	143:145	196:205	241:245	222:222
32	5	Br L	Vin	4/173	Br77	127:130	132:132	195:201	143:145	196:202	251:253	216:222
		Br L	Vin	4/173	Br79							
		Br L	Vin	4/173	Br76							
		Br L	Vin	4/173	Br78							
		Br L	Vin	4/173	Br80							
33	3	Br L	Vin	4/34	Br81	127:130	132:135	195:195	145:145	196:202	245:253	222:222
		Br L	Vin	4/34	Br83							
		Br L	Vin	4/34	Br85							
34	2	Br L	Vin	4/34	Br82	130:130	132:132	195:195	143:143	205:205	241:251	210:222
		Br L	Vin	4/34	Br84							
35	4	Br L	Vin	2/21	Br87	124:130	132:132	195:195	145:147	202:208	251:251	222:222
		Br L	Vin	2/21	Br89							
		Br L	Vin	2/21	Br86							
		Br L	Vin	2/21	Br90							
36	1	Br L	Vin	2/21	Br88	130:130	132:135	195:201	143:143	202:205	251:251	222:222
37	1	Br L	Vin	6/65	Br92	124:127	129:132	195:195	143:145	196:202	241:251	222:222
38	1	Br L	Vin	6/65	Br94	124:127	129:132	195:195	143:145	196:205	241:251	222:222
39	1	Br R	RH	5/139	Br104	124:130	129:132	195:201	143:145	202:208	251:251	222:222
40	1	Br R	RH	4/124	Br106	124:127	132:132	195:201	145:147	202:202	241:253	219:222
41	3	Br R	RH	1/142	Br168	124:127	132:132	195:201	143:145	202:202	241:253	219:222
		Br R	RH	4/124	Br108							
		Br R	RH	6/127	Br113							
42	17	Br R	RH	3/135	Br119	124:130	129:129	195:195	145:145	196:208	241:251	222:222
		Br R	RH	4/86	Br129							
		Br R	RH	4/87	Br135							
		Br R	RH	4/87	Br132							
		Br R	RH	4/88	Br139							
		Br R	RH	4/88	Br136							
		Br R	RH	5/84	Br157							
		Br R	RH	5/84	Br158							
		Br R	RH	5/85	Br153							
		Br R	RH	5/85	Br154							
		Br R	RH	6/127	Br115							
		Br R	RH	6/127	Br112							
		Br R	RH	6/67	Br149							
		Br R	RH	6/67	Br148							
		Br R	RH	7/122	Br121							
		Br R	RH	7/122	Br123							
		Br R	RH	7/122	Br122							
43	1	Br R	RH	6/127	Br114	115:124	132:132	195:201	143:145	202:208	251:251	222:222
44	1	Br R	RH	3/135	Br116	124:130	129:129	195:195	145:145	196:208	241:251	219:222
45	1	Br R	RH	3/135	Br118	124:130	129:129	195:195	143:145	208:208	251:251	222:222
46	1	Br R	RH	7/122	Br124	124:130	129:129	195:195	155:155	196:208	241:251	222:222
47	1	Br R	RH	7/122	Br125	124:130	129:129	201:201	145:145	196:208	241:251	222:222
48	2	Br R	RH	4/86	Br126	127:130	132:132	195:195	145:145	205:205	251:253	222:222
		Br R	RH	4/86	Br130							
49	1	Br R	RH	4/86	Br127	127:130	132:132	195:213	143:145	202:208	245:253	219:222
50	1	Br R	RH	4/87	Br134	124:130	129:129	195:195	147:147	196:208	241:251	222:222
51	1	Br R	RH	4/88	Br138	130:130	132:132	201:201	143:145	202:208	245:251	222:222
52	1	Br R	RH	6/66	Br142	127:130	132:132	195:198	143:145	202:205	251:251	222:222
53	1	Br R	RH	6/66	Br143	124:136	129:129	195:195	145:147	202:205	251:251	222:222
54	1	Br R	RH	6/66	Br144	121:127	132:132	195:195	145:147	205:208	251:253	219:222
55	1	Br R	RH	6/66	Br145	121:127	132:132	195:195	145:147	205:208	251:253	222:222
56	4	Br R	RH	5/53	Br161	124:127	129:132	195:195	143:145	202:205	251:253	216:222
		Br R	RH	5/53	Br163							
		Br R	RH	5/53	Br164							
		Br R	RH	6/67	Br150							
57	1	Br R	RH	5/85	Br152	124:130	129:129	195:195	147:147	202:208	241:251	222:222
58	1	Br R	RH	5/84	Br156	124:124	129:132	195:195	143:145	196:202	241:251	219:222
59	1	Br R	RH	5/84	Br159	127:130	129:132	195:213	143:145	202:208	245:253	219:222
60	1	Br R	RH	5/84	Br160	124:130	129:129	195:195	145:145	208:208	241:251	222:222
61	1	Br R	RH	5/53	Br162	124:127	132:132	195:195	145:145	202:208	251:251	210:210
62	1	Br R	RH	5/53	Br165	124:127	129:132	195:195	143:145	202:205	251:251	222:222
63	1	Br R	RH	1/142	Br167	124:127	123:132	195:201	143:145	202:202	241:241	219:222
64	2	Br R	RH	1/142	Br169	124:127	132:132	195:201	143:145	202:205	241:251	222:222

MLG	n	location	hostplant	plant code	sample code	PhyIII55	PhyIII30	PhyIII36	DV8	Dvit6	DVSSR4	DV4
		Br R	RH	1/142	Br170							
65	1	Br R	RH	1/79	Br171	124:127	132:132	195:195	143:145	187:190	233:233	201:201
66	2	Br R	RH	1/79	Br173	124:127	132:132	195:195	143:145	202:205	251:251	222:222
		Br R	RH	1/79	Br175							
67	5	Br R	RH	4/173	Br177	124:127	132:132	195:195	145:147	202:208	251:253	216:222
		Br R	RH	4/173	Br179							
		Br R	RH	4/173	Br176							
		Br R	RH	4/173	Br178							
		Br R	RH	4/173	Br180							
68	1	Br R	RH	8/89	Br181	127:130	132:132	195:195	145:145	196:202	251:253	216:222
69	1	Br R	RH	8/89	Br184	124:127	132:132	195:195	145:145	202:208	245:251	222:222
70	1	Br R	RH	8/89	Br185	127:130	132:132	195:201	143:145	202:208	251:251	210:222
71	4	Pf L	Vin	2/28	Pf186	127:130	129:132	195:204	145:145	205:208	251:253	216:219
		Pf L	Vin	2/28	Pf187							
		Pf L	Vin	2/28	Pf188							
		Pf L	Vin	2/28	Pf189							
72	3	Pf L	Vin	3/1	Pf190	127:130	132:132	195:195	145:145	208:208	251:253	216:228
		Pf L	Vin	3/1	Pf191							
		Pf L	Vin	3/1	Pf192							
73	3	Pf L	Vin	3/1	Pf193	127:130	132:132	195:195	147:147	208:208	251:253	216:228
		Pf L	Vin	3/1	Pf194							
		Pf L	Vin	3/1	Pf195							
74	1	Pf L	Vin	2/1	Pf196	127:130	132:132	195:207	143:143	205:208	251:253	216:222
75	3	Pf L	Vin	2/1	Pf198	130:130	129:132	195:207	143:145	202:208	251:253	216:228
		Pf L	Vin	2/1	Pf199							
		Pf L	Vin	2/1	Pf200							
76	1	Pf L	Vin	3/32	Pf201	127:130	129:132	195:195	145:147	205:205	251:253	222:222
77	1	Pf L	Vin	3/35	Pf202	127:127	120:132	198:204	143:145	208:208	251:253	222:222
78	1	Pf L	Vin	6/55	Pf209	127:130	132:132	195:207	145:145	205:205	251:251	219:228
79	1	Pf L	Vin	7/4	Pf210	124:127	132:132	195:207	143:147	202:208	251:251	222:228
80	3	Pf L	Vin	7/4	Pf212	124:127	132:132	195:207	143:145	202:208	251:251	222:228
		Pf L	Vin	7/4	Pf213							
		Pf L	Vin	7/4	Pf215							
81	1	Pf L	Vin	7/4	Pf214	127:127	132:132	195:207	143:145	202:208	251:251	222:228
82	1	Pf L	Vin	9/21	Pf216	124:130	132:132	195:207	143:145	202:205	251:253	222:222
83	5	Pf L	Vin	9/21	Pf217	127:130	132:132	195:207	143:143	202:205	251:251	222:222
		Pf R	RH	3/32	Pf225							
		Pf R	RH	3/32	Pf226							
		Pf R	RH	3/35	Pf233							
		Pf R	RH	7/4	Pf267							
84	1	Pf L	Vin	9/21	Pf218	124:130	132:132	195:207	143:145	202:205	251:253	216:222
85	1	Pf R	RH	2/28	Pf220	127:130	132:132	204:207	145:147	202:208	245:251	216:222
86	2	Pf R	RH	2/28	Pf221	127:130	132:132	204:207	143:145	202:208	245:251	216:222
		Pf R	RH	2/28	Pf222							
87	1	Pf R	RH	2/28	Pf223	127:130	132:132	195:207	143:143	202:202	251:251	222:222
88	1	Pf R	RH	2/28	Pf224	130:130	132:132	195:207	143:143	202:202	251:251	222:228
89	1	Pf R	RH	3/35	Pf232	124:127	129:132	195:195	143:147	208:208	251:251	219:222
90	2	Pf R	RH	3/35	Pf234	130:130	132:132	195:195	143:143	202:205	251:251	222:228
		Pf R	RH	5/41	Pf262							
91	1	Pf R	RH	3/35	Pf235	127:127	129:132	195:204	143:145	205:205	251:253	216:219
92	3	Pf R	RH	3/1	Pf236	127:130	129:132	195:195	145:147	205:208	251:251	219:228
		Pf R	RH	7/4	Pf265							
		Pf R	RH	7/4	Pf266							
93	4	Pf R	RH	3/1	Pf237	127:130	129:132	195:195	143:145	205:208	251:251	219:228
		Pf R	RH	3/1	Pf240							
		Pf R	RH	5/19	Pf249							
		Pf R	RH	7/4	Pf268							
94	2	Pf R	RH	3/1	Pf238	130:130	129:132	195:198	145:145	205:205	251:253	222:228
		Pf R	RH	3/1	Pf239							
95	4	Pf R	RH	2/1	Pf241	121:124	132:132	195:195	143:147	202:205	251:251	222:222
		Pf R	RH	2/1	Pf242							
		Pf R	RH	2/1	Pf244							
		Pf R	RH	2/1	Pf245							
96	1	Pf R	RH	2/1	Pf243	121:124	132:132	195:195	145:149	202:205	251:251	222:222
97	3	Pf R	RH	6/55	Pf246	127:127	129:132	195:195	145:145	202:208	251:251	222:222
		Pf R	RH	6/55	Pf247							
		Pf R	RH	6/55	Pf248							
98	2	Pf R	RH	5/33	Pf254	127:130	132:132	195:207	143:145	202:202	251:251	222:222
		Pf R	RH	5/33	Pf257							
99	1	Pf R	RH	5/33	Pf256	127:130	132:132	195:207	145:145	202:202	251:251	222:222
100	1	Pf R	RH	5/33	Pf258	127:130	132:132	195:195	143:145	202:208	251:251	219:228
101	1	Pf R	RH	5/41	Pf263	127:127	132:132	195:198	143:143	202:205	241:251	222:222

MLG	n	location	hostplant	plant code	sample code	PhyIII55	PhyIII30	PhyIII36	DV8	Dvit6	DVSSR4	DV4
102	1	Pf R	RH	7/4	Pf264	124:130	129:132	195:195	143:145	202:202	251:251	222:222
103	3	Ih T	RH	1/37	Ih271	124:127	129:132	195:195	143:145	202:208	251:251	216:216
		Ih T	RH	1/37	Ih272							
		Ih T	RH	1/37	Ih273							
104	2	Ih T	RH	2/24	Ih274	124:124	120:132	195:195	143:147	205:205	251:253	222:222
		Ih T	RH	2/24	Ih276							
105	4	Ih T	RH	3/19	Ih277	121:124	132:132	195:207	145:145	205:205	251:251	222:222
		Ih T	RH	3/19	Ih278							
		Ih T	RH	3/19	Ih280							
		Ih T	RH	3/19	Ih281							
106	1	Ih T	RH	4/69	Ih282	124:127	132:132	195:195	145:145	202:208	251:251	210:228
107	1	Ih T	RH	5/7	Ih285	124:130	132:132	195:195	143:143	205:208	241:251	210:216
108	1	Ih T	RH	6/65	Ih291	124:130	132:132	195:198	143:145	205:208	251:251	216:216
109	2	Ih T	RH	6/65	Ih292	124:130	132:132	195:195	143:147	208:208	251:251	210:222
		Ih L	Vin	18/52	Ih344							
110	1	Ih T	RH	6/65	Ih293	130:130	129:132	195:195	143:147	202:208	251:253	216:216
111	1	Ih T	RH	6/65	Ih294	127:127	132:132	195:207	143:149	202:205	245:251	216:228
112	1	Ih T	RH	6/65	Ih295	124:130	129:132	195:204	143:145	202:205	251:251	210:219
113	1	Ih T	RH	7/3	Ih296	124:130	129:132	195:195	143:147	205:208	251:251	216:216
114	1	Ih T	RH	7/3	Ih297	124:127	132:132	195:195	143:145	208:208	245:251	222:222
115	1	Ih T	RH	7/3	Ih298	124:130	123:132	195:195	145:145	202:202	251:251	222:222
116	1	Ih T	RH	7/3	Ih299	130:130	129:132	195:195	149:149	202:208	251:251	219:222
117	1	Ih T	RH	7/3	Ih300	130:130	129:132	195:195	147:147	202:208	251:251	219:222
118	1	Ih L	Vin	15/17	Ih328	127:130	132:132	207:207	143:143	202:202	251:251	210:228
119	1	Ih L	Vin	15/17	Ih329	124:130	132:132	195:195	143:149	202:202	251:251	219:222
120	1	Ih L	Vin	15/17	Ih330	124:130	132:132	195:195	145:145	205:205	251:251	216:222
121	1	Ih L	Vin	16/41	Ih331	121:130	132:132	195:210	143:149	202:208	251:253	219:222
122	1	Ih L	Vin	16/41	Ih332	121:130	123:132	195:210	143:147	202:208	251:253	219:222
123	1	Ih L	Vin	16/41	Ih333	130:130	132:132	195:195	145:145	202:208	251:251	216:222
124	1	Ih L	Vin	16/41	Ih334	124:127	132:132	195:213	143:145	205:205	251:253	210:222
125	1	Ih L	Vin	16/41	Ih335	124:130	132:132	195:198	145:145	208:208	251:253	210:222
126	1	Ih L	Vin	17/26	Ih336	127:127	132:132	195:210	143:147	208:208	251:251	210:219
127	1	Ih L	Vin	17/26	Ih340	124:127	129:132	195:195	145:147	208:208	251:253	210:219
128	1	Ih L	Vin	18/52	Ih341	124:130	129:132	195:210	143:147	208:208	251:251	210:216
129	2	Ih L	Vin	9/21	Ih360	130:130	132:132	195:195	145:147	202:202	251:251	222:222
		Ih L	Vin	18/52	Ih342							
130	3	Ih T	RH	10/52	Ih311	130:130	132:132	195:195	145:145	205:208	251:251	222:222
		Ih T	RH	9/18	Ih306							
		Ih L	Vin	18/52	Ih343							
131	1	Ih L	Vin	18/52	Ih345	124:127	132:132	195:207	145:147	202:208	251:251	219:222
132	1	Ih L	Vin	19/34	Ih348	127:130	132:132	195:213	143:145	202:208	251:253	210:216
133	2	Ih L	Vin	10/26	Ih363	124:127	132:132	207:213	145:145	202:208	251:251	210:228
		Ih L	Vin	19/34	Ih349							
134	1	Ih L	Vin	8/31	Ih352	124:127	132:132	195:207	147:147	202:208	251:253	210:219
135	1	Ih L	Vin	8/31	Ih353	127:130	132:132	195:207	145:147	208:208	251:251	210:222
136	1	Ih L	Vin	9/21	Ih357	121:130	132:132	195:195	143:145	205:208	245:251	210:219
137	1	Ih L	Vin	9/21	Ih358	124:130	129:132	195:195	145:147	208:208	251:253	216:222
138	1	Ih T	RH	8/65	Ih302	121:124	132:132	195:195	145:147	202:208	251:253	222:222
139	1	Ih T	RH	8/65	Ih303	127:130	132:132	195:210	143:145	208:208	251:251	222:228
140	2	Ih T	RH	8/65	Ih305	124:127	132:132	195:195	145:145	196:208	251:251	216:219
		Ih T	RH	9/18	Ih307							
141	1	Ih T	RH	9/18	Ih308	121:130	132:132	195:207	145:145	202:208	251:251	210:222
142	1	Ih T	RH	9/18	Ih309	124:130	132:132	195:210	147:147	202:205	251:251	210:219
143	1	Ih T	RH	9/18	Ih310	130:132	123:132	195:195	145:147	205:205	251:251	210:222
144	1	Ih T	RH	10/52	Ih312	130:130	120:132	195:207	145:145	202:208	251:251	210:222
145	1	Ih L	Vin	9/21	Ih359	121:124	132:132	195:195	145:145	208:208	251:251	210:228
146	1	Ih L	Vin	10/26	Ih361	127:127	132:132	195:207	145:147	202:202	251:253	210:228
147	1	Ih L	Vin	10/26	Ih362	124:130	132:132	195:207	145:147	202:205	251:251	216:219
148	1	Ih L	Vin	10/26	Ih364	130:130	132:132	195:204	143:145	205:208	251:251	216:222
149	1	Ih L	Vin	10/26	Ih365	127:130	132:132	195:195	145:147	208:208	251:251	210:216
150	1	Ih L	Vin	10/26	Ih366	124:127	129:132	195:210	145:145	202:205	251:253	210:210
151	1	Ih L	Vin	10/26	Ih367	124:130	129:132	195:195	143:145	202:208	251:251	210:216
152	1	Ih R	RH	13/9	Ih378	127:130	132:132	195:195	143:147	205:208	251:253	210:222
153	1	Ih R	RH	13/9	Ih379	124:124	132:132	195:207	143:145	205:208	251:251	222:222
154	4	Ih R	RH	13/9	Ih380	124:124	132:132	195:210	143:145	205:208	251:251	216:219
		Ih R	RH	18/63	Ih386							
		Ih R	RH	18/63	Ih387							
		Ih R	RH	18/63	Ih388							
155	3	Ih R	RH	18/63	Ih389	124:127	129:132	195:195	145:147	208:208	251:251	219:228
		Ih R	RH	19/24	Ih395							
		Ih R	RH	21/14	Ih405							
156	1	Ih R	RH	18/63	Ih390	124:130	132:132	195:195	143:145	205:208	245:251	210:222

MLG	n	location	hostplant	plant code	sample code	PhyIII55	PhyIII30	PhyIII36	DV8	Dvit6	DVSSR4	DV4
157	1	Ih R	RH	19/24	Ih394	124:127	129:132	195:195	143:143	205:208	245:251	219:222
158	3	Ih R	RH	20/3	Ih396	124:127	132:132	195:195	145:147	208:208	251:251	219:228
		Ih R	RH	21/14	Ih402							
		Ih R	RH	21/14	Ih404							
159	2	Ih R	RH	20/3	Ih399	124:130	132:132	195:210	143:145	208:208	251:251	219:222
		Ih R	RH	21/14	Ih401							
160	1	Ih R	RH	21/14	Ih412	124:124	132:132	195:195	143:147	205:208	251:251	219:222
161	1	Ih R	RH	22/38	Ih418	121:127	132:132	195:195	143:143	202:208	241:253	216:222
162	1	Ih R	RH	22/38	Ih419	124:136	123:132	195:201	143:145	202:208	251:251	210:222
163	1	Ih R	RH	22/38	Ih420	130:130	132:132	195:207	143:143	202:208	241:253	216:222
164	1	Ih R	RH	23/47	Ih425	124:130	120:120	195:204	143:143	202:208	241:253	216:222
165	1	Ih R	RH	23/47	Ih429	124:127	132:132	195:207	143:145	202:208	251:251	219:222
166	1	Ih R	RH	24/62	Ih434	124:124	132:132	195:198	143:145	202:208	251:251	219:222
167	1	Ih R	RH	24/62	Ih435	127:127	120:132	195:207	143:145	202:208	251:251	219:222
168	1	Ih R	RH	25/16	Ih438	124:124	120:132	195:195	143:145	202:208	251:251	219:222
169	2	Ih R	RH	25/16	Ih439	124:127	132:132	195:207	143:145	205:208	251:253	216:222
		Ih R	RH	25/16	Ih440							
170	1	Ih T	RH	10/52	Ih314	124:127	132:132	195:207	143:149	202:205	251:251	222:222
171	1	Ih T	RH	10/52	Ih315	127:130	132:132	195:195	145:147	202:205	249:249	207:216
172	1	Ih L	Vin	10/26	Ih368	124:127	129:132	195:195	145:147	202:208	251:251	210:222
173	1	Ih L	Vin	10/26	Ih369	124:127	132:132	195:207	145:145	208:208	251:251	210:222
174	1	Ih L	Vin	10/26	Ih370	124:130	129:132	195:195	143:143	202:205	251:251	219:222
175	1	Ih L	Vin	11/41	Ih371	127:130	132:132	195:198	143:145	208:208	245:251	216:222
176	1	Ih L	Vin	11/41	Ih372	124:130	132:132	195:195	147:149	202:205	251:251	210:222
177	1	Ih R	RH	25/16	Ih441	124:124	132:132	195:195	145:145	205:208	251:251	210:222
178	1	Ih R	RH	25/16	Ih442	127:130	132:132	195:213	143:143	205:208	241:251	216:216
179	1	Ih R	RH	26/40	Ih443	124:124	129:132	195:213	143:143	205:208	241:251	216:216
180	1	Ih R	RH	26/40	Ih444	124:127	132:132	195:198	147:147	205:208	245:251	222:222
181	1	Ih R	RH	26/40	Ih445	124:130	132:132	198:207	143:143	205:208	241:251	216:216
182	1	Ih R	RH	26/40	Ih446	124:127	132:132	195:198	143:143	205:208	241:251	216:216
183	1	Ih L	Vin	11/41	Ih373	124:130	129:132	195:195	143:145	202:202	251:253	210:222
184	1	Ih R	RH	27/28	Ih447	124:130	132:132	195:213	145:145	202:208	251:251	210:219
185	1	Ih R	RH	27/28	Ih450	124:130	132:132	195:213	143:143	199:202	249:249	207:213
186	1	Ih L	Vin	11/41	Ih375	127:130	132:132	195:195	143:147	208:208	251:251	210:219
187	1	Ih L	Vin	13/14	Ih376	124:130	132:132	195:213	145:145	205:208	251:251	210:222
188	1	Ih T	RH	11/64	Ih316	127:127	129:132	195:195	145:147	205:208	251:251	222:228
189	1	Ih T	RH	11/64	Ih317	124:124	129:132	195:195	145:145	202:205	251:251	216:216
190	1	Ih T	RH	11/64	Ih318	121:124	129:132	198:198	143:145	202:208	251:251	210:216
191	1	Ih T	RH	11/64	Ih319	121:124	132:132	195:210	145:145	205:208	251:251	222:222
192	1	Ih T	RH	11/64	Ih320	124:130	129:129	195:207	145:145	202:205	251:251	210:210
193	1	Ih T	RH	12/37	Ih322	130:133	132:135	195:195	143:147	202:208	245:251	216:222
194	1	Ih T	RH	12/37	Ih323	127:127	132:132	195:198	143:145	202:205	245:251	216:222
195	1	Ih T	RH	12/37	Ih324	124:130	132:132	195:195	143:143	208:208	251:251	216:216
196	1	Ih T	RH	12/37	Ih325	124:136	129:132	192:195	147:149	205:208	253:253	216:222
197	1	Ih T	RH	14/43	Ih326	127:130	132:132	195:195	147:149	202:205	251:253	210:219
198	1	Ih T	RH	14/43	Ih327	124:127	129:132	195:195	145:149	202:208	251:251	222:228
199	1	Ba T	RH	1/8	Ba451	124:127	129:132	195:195	143:143	202:202	245:245	210:216
200	1	Ba T	RH	1/8	Ba453	124:130	132:132	195:207	143:143	202:202	245:245	210:216
201	1	Ba T	RH	1/8	Ba454	124:124	129:132	195:210	143:143	202:202	245:245	210:216
202	1	Ba T	RH	1/8	Ba455	124:124	129:132	195:210	147:147	202:202	253:253	219:222
203	1	Ba T	RH	2/34	Ba457	124:124	129:132	195:210	143:145	202:202	251:251	210:222
204	1	Ba T	RH	2/34	Ba458	124:124	129:129	195:198	147:147	202:208	245:253	210:216
205	1	Ba T	RH	2/34	Ba459	124:124	129:132	195:195	145:147	202:208	251:253	210:219
206	1	Ba T	RH	2/34	Ba460	124:124	129:132	195:195	143:145	202:205	245:251	210:213
207	1	Ba L	FR	3/15	Ba461	124:124	129:132	195:195	145:147	202:208	253:253	222:222
208	1	Ba L	FR	3/15	Ba462	124:124	129:132	195:195	145:147	202:208	251:253	210:222
209	1	Ba L	FR	3/15	Ba464	124:136	129:132	195:195	149:149	205:208	251:255	210:216
210	1	Ba L	FR	4/26	Ba467	124:124	132:132	195:195	143:145	202:208	251:251	219:222
211	1	Ba L	FR	5/23	Ba472	127:127	132:132	195:195	143:147	202:202	251:251	219:222
212	6	Ba T	RH	10/2	Ba496	121:127	120:132	195:198	143:145	202:205	241:245	210:216
		Ba T	RH	10/2	Ba497							
		Ba T	RH	10/2	Ba498							
		Ba L	FR	6/42	Ba480							
		Ba T	RH	9/22	Ba491							
		Ba T	RH	9/22	Ba492							
213	1	Ba L	FR	7/6	Ba481	127:127	132:132	195:210	143:147	202:208	251:251	210:222
214	1	Ba L	FR	7/6	Ba482	124:127	120:132	195:195	143:145	202:202	245:253	210:219
215	1	Ba L	FR	7/6	Ba484	124:130	132:132	195:195	145:147	202:208	251:251	210:222
216	1	Ba L	FR	8/43	Ba488	124:127	132:132	195:195	143:147	202:202	241:253	222:222
217	1	Ba L	FR	8/43	Ba489	127:127	129:132	195:195	147:147	202:202	245:251	213:216
218	1	Ba L	FR	8/43	Ba490	124:127	120:132	204:210	143:145	196:202	251:251	219:219
219	1	Ba T	RH	10/2	Ba499	124:124	129:132	198:213	145:145	202:208	251:253	210:222

MLG	n	location	hostplant	plant code	sample code	PhyIII55	PhyIII30	PhyIII36	DV8	Dvit6	DVSSR4	DV4
220	1	Ba T	RH	10/2	Ba500	124:130	132:132	195:207	143:145	202:208	251:251	219:222
221	1	Ba L	FR	11/16	Ba501	124:124	132:132	195:195	147:147	202:202	245:253	222:222
222	1	Ba L	FR	11/16	Ba504	121:127	129:132	195:195	145:147	202:208	251:251	222:222
223	1	Ba L	FR	11/16	Ba505	121:130	132:132	195:195	145:145	202:208	251:251	210:222
224	2	Ba L	FR	12/65	Ba506	130:130	120:132	195:195	143:147	202:205	251:253	216:222
		Ba L	FR	12/65	Ba507							
225	1	Ba L	FR	12/65	Ba508	124:127	132:132	195:195	147:147	202:202	251:251	222:222
226	1	Ba L	FR	12/65	Ba510	127:127	120:132	195:195	145:147	202:208	251:251	222:222
227	1	Ba L	FR	13/45	Ba512	124:124	132:132	195:210	145:147	205:208	251:251	222:222
228	1	Ba L	FR	13/45	Ba513	127:130	120:132	195:207	143:147	202:208	245:251	216:222
229	4	Ba L	FR	14/21	Ba514	124:127	129:132	195:207	143:145	202:208	251:255	219:222
		Ba L	FR	14/21	Ba515							
		Ba L	FR	14/21	Ba516							
		Ba L	FR	14/21	Ba517							
230	2	Ba L	FR	15/38	Ba519	127:127	132:132	195:195	145:145	202:202	245:253	210:222
		Ba L	FR	15/38	Ba520							
231	1	Ba L	FR	16/14	Ba521	124:124	132:132	195:195	145:147	202:202	251:251	222:222
232	1	Ba L	FR	16/14	Ba522	121:124	132:132	195:201	145:145	202:205	245:251	210:216
233	3	Ba T	RH	17/82	Ba523	124:127	132:132	195:195	145:147	202:202	251:251	210:219
		Ba T	RH	17/82	Ba524							
		Ba T	RH	17/82	Ba526							
234	1	Ba L	Vin	18/36	Ba528	121:124	120:132	195:195	145:145	202:202	251:251	210:219
235	1	Ba L	Vin	18/36	Ba529	124:127	120:132	195:207	145:145	202:208	245:245	210:222
236	1	Ba L	Vin	18/36	Ba532	121:124	132:132	195:198	145:145	202:208	251:251	210:222
237	1	Ba L	Vin	20/41	Ba536	121:124	132:132	195:198	143:143	202:208	251:251	210:222
238	2	Ba L	FR	21/79	Ba537	124:127	129:132	195:195	145:147	202:208	245:251	210:210
		Ba L	FR	21/79	Ba540							
239	1	Ba L	FR	21/79	Ba538	124:127	132:132	195:195	143:147	202:202	251:251	222:222
240	1	Ba L	FR	21/79	Ba539	124:127	129:129	195:195	145:147	202:208	245:251	210:210
241	1	Ba L	FR	22/15	Ba544	124:127	132:132	195:195	145:147	202:202	251:251	219:222
242	1	Ba L	FR	22/15	Ba545	124:130	132:132	195:207	143:143	208:208	251:251	222:222
243	1	Ba L	FR	22/15	Ba546	127:127	120:132	195:198	143:145	202:202	251:253	210:222
244	1	Ba L	FR	23/9	Ba547	121:124	132:132	195:198	143:143	202:208	251:251	222:222
245	1	Ba L	FR	23/9	Ba548	121:121	120:129	195:198	143:147	196:202	251:253	210:222
246	1	Ba L	FR	23/9	Ba549	121:124	132:132	195:198	145:145	202:208	251:251	222:222
247	1	Ba L	FR	24/82	Ba551	127:130	132:132	195:195	145:147	202:208	251:251	222:222
248	1	Ba L	FR	24/82	Ba552	121:127	120:132	195:210	147:147	196:202	251:251	210:222
249	1	Ba L	FR	25/64	Ba556	124:127	132:132	195:195	145:147	202:202	245:251	210:216
250	1	Ba L	FR	25/64	Ba557	124:127	132:132	195:210	145:145	196:202	251:251	219:222
251	1	Ba L	FR	25/64	Ba558	124:124	129:132	195:198	145:147	202:208	251:255	219:222
252	1	Ba L	FR	25/64	Ba560	124:127	132:132	195:198	143:147	202:202	245:251	210:222
253	1	Ba L	FR	26/73	Ba561	124:127	132:132	195:201	143:145	205:208	251:251	210:219
254	1	Ba L	FR	26/73	Ba563	127:127	120:132	195:198	143:147	202:205	251:251	210:222
255	1	Ba L	FR	26/73	Ba564	127:127	132:132	195:195	147:149	205:208	251:251	210:222
256	1	Ba R	RH	18/36	Ba574	124:130	132:132	195:195	143:145	202:202	251:253	222:222
257	1	Ba R	RH	18/36	Ba577	124:130	132:132	195:195	145:145	202:202	251:253	222:222
258	1	Ba R	RH	18/36	Ba578	124:127	120:120	195:195	145:145	208:208	251:255	216:216
259	1	Ba R	RH	21/79	Ba579	124:127	129:132	195:195	143:145	202:208	245:251	210:210
260	1	Ba R	RH	21/79	Ba583	124:124	120:132	195:195	145:147	202:202	245:245	216:216
261	1	Ba R	RH	22/15	Ba584	127:127	129:132	195:195	145:147	202:202	251:253	210:222
262	1	Ba R	RH	27/41	Ba585	121:127	132:132	195:195	145:147	202:208	245:251	216:222
263	1	Ba R	RH	27/41	Ba590	121:127	132:132	195:195	143:145	202:208	245:251	216:222
264	3	Br L	Vin	31/19	Br603	127:130	132:132	195:195	143:145	202:202	251:253	222:222
		Br L	Vin	31/19	Br604							
		Br L	Vin	31/19	Br605							
265	2	ELP2	FR	K1	B2K652	124:127	132:132	195:207	145:145	202:205	241:251	219:222
		ELP2	FR	K1	B2K653							
266	9	ELP2	FR	K1	B2K656	115:124	129:132	195:195	143:145	202:205	241:241	222:222
		ELP2	FR	K1	B2K658							
		ELP2	FR	K1	B2K659							
		ELP2	FR	K1	B2K660							
		ELP2	FR	K1	B2K661							
		ELP2	FR	K1	B2K662							
		ELP2	FR	K1	B2K663							
		ELP2	FR	K1	B2K664							
		ELP2	FR	K1	B2K665							
267	9	ELP2	FR	K3	B2K699	124:127	120:132	195:195	143:145	205:205	241:251	219:222
		ELP2	FR	K3	B2K706							
		OLP2	FR	O	B2K666							
		OLP2	FR	O	B2K667							
		OLP2	FR	O	B2K668							
		OLP2	FR	O	B2K669							

MLG	n	location	hostplant	plant code	sample code	PhyIII55	PhyIII30	PhyIII36	DV8	Dvit6	DVSSR4	DV4
		OLP2	FR	O	B2K670							
		OLP2	FR	O	B2K671							
		OLP2	FR	O	B2K672							
268	9	ELP2	FR	K3	B2K708	124:127	132:132	195:195	143:145	202:205	251:253	216:222
		OLP2	FR	O	B2K673							
		OLP2	FR	O	B2K674							
		OLP2	FR	O	B2K675							
		OLP2	FR	O	B2K676							
		OLP2	FR	O	B2K678							
		OLP2	FR	O	B2K679							
		OLP2	FR	O	B2K680							
		OLP2	FR	O	B2K710							
269	1	OLP2	FR	O	B2K677	124:127	132:132	195:195	143:145	202:208	253:253	216:222
270	1	ELP2	FR	K2	B2K681	115:124	132:132	195:195	143:145	205:208	251:253	216:222
271	13	ELP2	FR	K2	B2K682	115:124	132:132	195:195	143:145	205:208	251:253	210:222
		ELP2	FR	K2	B2K683							
		ELP2	FR	K2	B2K684							
		ELP2	FR	K2	B2K685							
		ELP2	FR	K2	B2K686							
		ELP2	FR	K2	B2K687							
		ELP2	FR	K2	B2K688							
		ELP2	FR	K2	B2K689							
		ELP2	FR	K2	B2K690							
		ELP2	FR	K2	B2K691							
		ELP2	FR	K2	B2K692							
		ELP2	FR	K2	B2K693							
		ELP2	FR	K2	B2K694							
272	1	ELP2	FR	K3	B2K696	115:124	132:132	195:195	145:145	208:208	253:253	216:222
273	1	OLP2	FR	O	B2K709	124:130	132:132	195:195	143:145	202:208	253:253	216:222
274	14	ELP1	FR	K1	B1K711	127:130	120:132	195:195	145:147	202:208	253:253	222:222
		ELP1	FR	K1	B1K713							
		ELP1	FR	K1	B1K714							
		ELP1	FR	K1	B1K715							
		ELP1	FR	K1	B1K716							
		ELP1	FR	K1	B1K717							
		ELP1	FR	K1	B1K718							
		ELP1	FR	K1	B1K719							
		ELP1	FR	K1	B1K720							
		ELP1	FR	K1	B1K721							
		ELP1	FR	K1	B1K722							
		ELP1	FR	K1	B1K723							
		ELP1	FR	K1	B1K724							
		ELP1	FR	K1	B1K725							
275	7	ELP1	FR	K2	B1K726	124:127	132:132	195:195	145:147	202:202	251:253	210:222
		ELP1	FR	K2	B1K727							
		ELP1	FR	K2	B1K728							
		ELP1	FR	K2	B1K733							
		ELP1	FR	K2	B1K734							
		ELP1	FR	K2	B1K735							
		ELP1	FR	K2	B1K877							
276	6	ELP1	FR	K2	B1K729	121:124	132:132	195:207	143:145	202:208	251:251	222:222
		ELP1	FR	K2	B1K730							
		ELP1	FR	K2	B1K731							
		ELP1	FR	K2	B1K732							
		ELP1	FR	K2	B1K875							
		ELP1	FR	K2	B1K876							
277	1	OLP1	FR	O	B1K736	127:127	129:132	198:213	145:147	202:202	251:253	222:222
278	5	OLP1	FR	O	B1K737	127:127	129:132	198:213	143:145	202:202	251:253	222:222
		OLP1	FR	O	B1K738							
		OLP1	FR	O	B1K739							
		OLP1	FR	O	B1K750							
		OLP1	FR	O	B1K751							
279	1	OLP1	FR	O	B1K746	127:127	129:132	198:213	143:145	202:202	251:253	216:225
280	3	OLP1	FR	O	B1K747	127:127	129:132	198:213	143:145	202:202	251:253	210:222
		OLP1	FR	O	B1K752							
		OLP1	FR	O	B1K757							
281	1	OLP1	FR	O	B1K748	127:127	129:132	198:213	143:145	202:202	251:253	219:222
282	1	OLP1	FR	O	B1K749	127:127	129:132	198:213	143:145	202:202	251:253	213:216
283	2	OLP1	FR	O	B1K753	127:127	129:132	198:213	143:145	202:202	251:253	210:219
		OLP1	FR	O	B1K759							
284	2	OLP1	FR	O	B1K754	127:127	129:132	198:213	143:145	202:202	251:253	216:222
		OLP1	FR	O	B1K755							

MLG	n	location	hostplant	plant code	sample code	PhyIII55	PhyIII30	PhyIII36	DV8	Dvit6	DVSSR4	DV4
285	1	OLP1	FR	O	B1K756	127:127	129:132	198:213	143:145	202:202	251:253	219:219
286	1	OLP1	FR	O	B1K760	127:127	129:132	198:213	143:145	202:202	251:253	216:219
287	1	ERP2	RH	K1	B2K761	124:127	132:132	195:195	145:147	202:205	251:253	210:222
288	1	ERP2	RH	K1	B2K762	124:127	132:132	195:195	145:147	202:205	251:253	222:222
289	1	ERP2	RH	K1	B2K763	124:127	132:132	195:207	145:145	196:205	251:251	219:228
290	1	ERP2	RH	K1	B2K764	124:124	132:132	198:207	145:147	205:208	251:253	219:228
291	1	ERP2	RH	K1	B2K766	124:127	132:132	195:198	143:143	205:205	253:253	219:228
292	1	ERP2	RH	K1	B2K767	124:127	132:132	195:207	145:145	196:205	251:251	210:210
293	1	ERP2	RH	K1	B2K768	127:130	132:132	195:195	145:145	208:208	251:253	219:228
294	1	ERP2	RH	K2	B2K771	124:124	129:132	195:195	143:145	208:208	251:251	219:222
295	1	ERP2	RH	K2	B2K772	115:121	132:132	195:195	143:143	202:208	251:253	219:222
296	1	ERP2	RH	K2	B2K773	124:127	132:132	195:195	143:145	202:208	251:251	210:222
297	5	ERP2	RH	K2	B2K774	121:124	132:132	195:198	145:147	205:208	251:253	219:228
		ERP2	RH	K2	B2K776							
		ERP2	RH	K2	B2K780							
		ERP2	RH	K2	B2K781							
		ERP2	RH	K2	B2K783							
298	1	ERP2	RH	K2	B2K775	121:121	132:132	195:198	143:145	208:208	251:251	219:222
299	2	ERP2	RH	K2	B2K777	121:124	132:132	195:198	145:147	205:208	251:253	210:222
		ERP2	RH	K2	B2K796							
300	1	ERP2	RH	K2	B2K779	121:127	132:132	195:195	145:147	202:208	251:253	210:222
301	1	ERP2	RH	K2	B2K782	121:127	132:132	195:195	143:145	202:205	241:251	219:228
302	1	ERP2	RH	K2	B2K784	127:130	132:132	195:207	145:145	202:208	251:251	210:222
303	6	ERP2	RH	K2	B2K785	121:124	132:132	195:198	145:147	205:208	251:253	222:222
		ERP2	RH	K2	B2K788							
		ERP2	RH	K2	B2K791							
		ERP2	RH	K2	B2K792							
		ERP2	RH	K2	B2K797							
		ERP2	RH	K2	B2K800							
304	2	ERP2	RH	K2	B2K786	121:124	132:132	195:198	145:147	205:208	251:253	219:222
		ERP2	RH	K3	B2K823							
305	1	ERP2	RH	K2	B2K787	124:124	132:132	195:195	145:147	205:208	253:253	222:222
306	2	ERP2	RH	K2	B2K789	127:130	132:132	195:195	143:145	202:208	241:241	222:222
		ERP2	RH	K3	B2K808							
307	1	ERP2	RH	K2	B2K790	127:130	132:132	195:207	145:145	202:208	251:251	216:222
308	1	ERP2	RH	K2	B2K793	115:121	132:132	195:195	143:143	202:208	251:253	222:222
309	1	ERP2	RH	K2	B2K798	121:124	132:132	195:198	145:147	205:208	253:253	216:222
310	1	ERP2	RH	K3	B2K801	124:127	132:132	195:195	143:145	202:208	251:251	219:219
311	7	ERP2	RH	K3	B2K802	124:127	129:132	195:198	143:143	202:205	245:253	222:222
		ERP2	RH	K3	B2K803							
		ERP2	RH	K3	B2K804							
		ERP2	RH	K3	B2K805							
		ERP2	RH	K3	B2K807							
		ERP2	RH	K3	B2K809							
		ERP2	RH	K3	B2K811							
312	1	ERP2	RH	K3	B2K806	124:127	132:132	195:195	143:145	202:208	251:251	216:228
313	1	ERP2	RH	K3	B2K812	127:130	132:132	195:195	143:145	202:208	241:241	210:222
314	2	ERP2	RH	K3	B2K813	124:127	132:132	195:207	145:145	205:208	251:251	222:222
		ERP2	RH	K3	B2K815							
315	1	ERP2	RH	K3	B2K814	124:127	132:132	195:207	145:145	205:208	251:251	222:228
316	1	ERP2	RH	K3	B2K816	124:127	129:132	195:207	143:143	202:205	245:253	222:222
317	1	ERP2	RH	K3	B2K818	124:130	132:132	195:207	143:145	202:205	241:251	216:228
318	1	ERP2	RH	K3	B2K819	124:127	129:132	195:198	143:143	202:205	245:253	216:219
319	1	ERP2	RH	K3	B2K820	124:127	129:132	195:198	143:143	202:205	245:253	210:219
320	1	ERP2	RH	K3	B2K821	124:127	129:132	195:198	143:143	202:205	245:253	210:222
321	1	ERP2	RH	K3	B2K822	124:127	129:132	195:198	143:143	202:205	245:253	216:228
322	1	ERP1	RH	K1	B1K825	124:124	132:132	195:207	143:145	202:208	245:255	222:222
323	2	ERP1	RH	K1	B1K826	127:127	132:132	195:207	143:145	202:208	251:255	222:222
		ERP1	RH	K1	B1K832							
324	1	ERP1	RH	K1	B1K829	124:127	132:132	195:207	145:147	202:208	241:255	222:222
325	2	ERP1	RH	K1	B1K830	124:130	120:132	195:195	145:147	202:202	251:253	222:222
		ERP1	RH	K1	B1K831							
326	1	ERP1	RH	K1	B1K833	124:127	132:132	195:195	145:147	202:202	245:251	222:222
327	1	ERP1	RH	K1	B1K834	127:127	120:120	195:195	145:147	202:202	251:253	222:222
328	2	ERP1	RH	K1	B1K835	121:127	132:132	195:195	143:143	202:208	245:251	222:222
		ERP1	RH	K1	B1K837							
329	1	ERP1	RH	K1	B1K836	124:127	132:132	195:195	147:147	202:202	245:253	222:222
330	1	ERP1	RH	K1	B1K838	127:127	120:132	195:195	145:145	202:205	253:253	222:222
331	1	ERP1	RH	K1	B1K839	124:124	132:132	195:195	143:145	202:205	251:253	210:222
332	2	ERP1	RH	K1	B1K840	124:127	132:132	195:207	145:147	202:208	241:255	219:222
		ERP1	RH	K2	B1K857							
333	1	ERP1	RH	K1	B1K841	127:127	120:132	195:195	145:147	202:202	245:243	210:210

MLG	n	location	hostplant	plant code	sample code	PhyIII55	PhyIII30	PhyIII36	DV8	Dvit6	DVSSR4	DV4
334	2	ERP1	RH	K1	B1K842	124:124	132:132	195:195	145:147	202:208	245:251	210:216
		ERP1	RH	K1	B1K843							
335	1	ERP1	RH	K1	B1K844	124:127	132:132	195:195	147:147	202:202	245:253	213:216
336	1	ERP1	RH	K1	B1K845	121:127	132:132	195:195	143:145	202:202	245:253	219:222
337	1	ERP1	RH	K1	B1K846	127:127	120:132	195:195	145:145	202:205	253:253	210:222
338	1	ERP1	RH	K1	B1K847	127:127	132:132	195:207	143:145	202:208	251:255	210:219
339	2	ERP1	RH	K1	B1K848	124:127	132:132	195:195	143:145	202:205	241:251	210:222
		ERP1	RH	K1	B1K849							
340	3	ERP1	RH	K2	B1K851	124:124	132:132	195:204	147:147	202:202	241:253	219:222
		ERP1	RH	K2	B1K859							
		ERP1	RH	K2	B1K866							
341	3	ERP1	RH	K2	B1K852	121:124	132:132	195:195	145:145	202:202	251:251	210:222
		ERP1	RH	K2	B1K867							
		ERP1	RH	K2	B1K871							
342	1	ERP1	RH	K2	B1K854	127:130	120:132	195:210	145:145	196:202	251:251	219:222
343	1	ERP1	RH	K2	B1K855	124:127	132:132	195:195	143:145	205:208	251:253	219:222
344	1	ERP1	RH	K2	B1K856	124:127	120:132	195:195	145:147	205:208	245:253	210:219
345	1	ERP1	RH	K2	B1K858	124:127	132:132	195:195	143:145	202:208	245:251	210:219
346	1	ERP1	RH	K2	B1K860	124:130	132:132	195:207	145:145	202:208	251:251	210:222
347	4	ERP1	RH	K2	B1K861	124:127	132:132	195:195	145:145	202:205	245:253	210:222
		ERP1	RH	K2	B1K864							
		ERP1	RH	K2	B1K865							
		ERP1	RH	K2	B1K872							
348	2	ERP1	RH	K2	B1K868	124:124	132:132	195:204	143:145	202:205	245:255	222:222
		ERP1	RH	K2	B1K874							
349	1	ERP1	RH	K2	B1K870	127:127	132:132	195:195	143:147	208:208	251:251	222:222
350	5	Pf L	Vin	6/62	Pf879	124:130	132:132	195:195	145:145	202:208	253:253	210:216
		Pf L	Vin	6/62	Pf880							
		Pf L	Vin	6/62	Pf881							
		Pf L	Vin	6/62	Pf882							
		Pf L	Vin	6/62	Pf883							
